# Spermatocyte injection into meiotic oocytes rescues diplotene, but not pachytene, arrest in azoospermic mutant mice

**DOI:** 10.1093/hropen/hoaf067

**Published:** 2025-10-22

**Authors:** Narumi Ogonuki, Toshiaki Hino, Yasuhiro Fujiwara, Yuki Osawa, Seiya Mizuno, Fumihiro Sugiyama, Tetsuo Kunieda, Junko Otsuki, Seiya Oura, Tamio Furuse, Yuki Okada, Masaru Tamura, Elena de la Casa-Esperon, Masahito Ikawa, Kimiko Inoue, Atsuo Ogura

**Affiliations:** Integrative Developmental Engineering Division, RIKEN BioResource Research Center, Tsukuba, Ibaraki, Japan; Department of Biological Sciences, Asahikawa Medical University, Asahikawa, Hokkaido, Japan; Laboratory of Pathology and Development, Institute for Quantitative Biosciences, The University of Tokyo, Bunkyo-ku, Tokyo, Japan; Master’s Program in Medical Sciences, Graduate School of Comprehensive Human Sciences, University of Tsukuba, Tsukuba, Ibaraki, Japan; Laboratory Animal Resource Center and Trans-border Medical Research Center, Faculty of Medicine, University of Tsukuba, Tsukuba, Ibaraki, Japan; Laboratory Animal Resource Center and Trans-border Medical Research Center, Faculty of Medicine, University of Tsukuba, Tsukuba, Ibaraki, Japan; Graduate School of Environmental Life, Natural Science and Technology, Okayama University, Okayama, Okayama, Japan; Hanabusa Women’s Clinic, Kobe, Hyogo, Japan; Department of Experimental Genome Research, Research Institute for Microbial Diseases, Osaka University, Suita, Osaka, Japan; Department of Molecular Biology, University of Texas Southwestern Medical Center, Dallas, TX, USA; Mouse Phenomics Division, RIKEN BioResource Research Center, Tsukuba, Ibaraki, Japan; Laboratory of Pathology and Development, Institute for Quantitative Biosciences, The University of Tokyo, Bunkyo-ku, Tokyo, Japan; Mouse Phenomics Division, RIKEN BioResource Research Center, Tsukuba, Ibaraki, Japan; Biomedicine Institute (IB) and Faculty of Pharmacy, University of Castilla-La Mancha and IDISCAM, Albacete, Spain; Department of Experimental Genome Research, Research Institute for Microbial Diseases, Osaka University, Suita, Osaka, Japan; Integrative Developmental Engineering Division, RIKEN BioResource Research Center, Tsukuba, Ibaraki, Japan; Educational Programs in Agro-Biological Resource Sciences, University of Tsukuba, Tsukuba, Ibaraki, Japan; Integrative Developmental Engineering Division, RIKEN BioResource Research Center, Tsukuba, Ibaraki, Japan

**Keywords:** fertilization, meiosis, oocyte, azoospermia, spermatocyte, mouse, mutation

## Abstract

**STUDY QUESTION:**

At which arrest stage can spermatocytes be rescued by injection into meiotic oocytes?

**SUMMARY ANSWER:**

In mice, spermatocytes arrested at the diplotene stage, but not at the pachytene stage, can resume meiosis within immature oocytes and support full-term embryonic development.

**WHAT IS KNOWN ALREADY:**

In mice, at least some of the spermatocyte arrest mutations can be overcome by injecting spermatocytes into immature oocytes.

**STUDY DESIGN, SIZE, DURATION:**

The study was carried out from October 2019 to April 2025. Adult azoospermic mice (at 4–26 weeks of age) from nine strains carrying spermatocyte arrest mutations were used as spermatocyte donors. Adult B6D2F1 females at 9–12 weeks of age were used as oocyte donors for spermatocyte injection. Adult ICR strain pseudopregnant females at 9–12 weeks of age were used as recipients for embryo transfer experiments.

**PARTICIPANTS/MATERIALS, SETTING, METHODS:**

The most advanced stage of spermatocytes from each mutant strain was assessed by chromosome spread analysis. These most advanced spermatocytes of each strain were injected into metaphase I (MI) oocytes. About half a volume of the ooplasm had been removed from the recipient oocytes to ensure more stable chromosome behaviours during meiosis. The spermatocyte-injected oocytes were allowed to mature *in vitro* to the metaphase II (MII) stage, and their ooplasm was refreshed with the ooplasm from intact MII oocytes. After activation with SrCl_2_, the reconstructed oocytes that reached the 2-cell stage were transferred into the oviducts of pseudopregnant females. On Day 19.5, recipient females were euthanized and their uteri were examined for live foetuses.

**MAIN RESULTS AND THE ROLE OF CHANCE:**

Based on spermatocyte spread analysis, sperm mutants were categorized into three classes: Class 1, arrest at mid-diplotene or later stage; Class 2, arrest at early diplotene stage; and Class 3, arrest at pachytene stage. All four Class 1 mutants could resume normal meiosis following injection into MI oocytes, as evidenced by births of normal offspring. Similarly, one of two Class 2 mutants could be rescued, but the other could not. By contrast, three Class 3 mutants did not support embryo development to term because of complete implantation failure, indicating that reconstructed embryos carried severe chromosomal aberrations.

**LARGE-SCALE DATA:**

N/A.

**LIMITATIONS, REASONS FOR CAUTION:**

The number of mutant strains examined was limited. Nevertheless, the findings were consistent: the more advanced the arrest stage of spermatocytes, the higher the likelihood of a successful rescue.

**WIDER IMPLICATIONS OF THE FINDINGS:**

In humans, a considerable proportion of spermatogenic arrest occurs at the primary spermatocyte stage. Spermatocyte injection might be an option to treat human male-factor infertility due to azoospermia in the future. However, numerous ethical and technical challenges remain to be addressed, and the reproductive physiological differences between mice and humans must be carefully taken into account.

**STUDY FUNDING/COMPETING INTEREST(S):**

This study was supported by Grants-in-Aid for Scientific Research (KAKENHI) from the Japan Society for the Promotion of Science to A.O. (grant number: JP19H05758), K.I. (grant number: 23H04956), M.I. (grant number: JP23K20043), and N.O. (grant number: 25H01372), and 2023 and 2025 grants of the University of Castilla-La Mancha for stays in foreign universities and research centres to E.C.-E. The authors declare that they have no conflicts of interest.

WHAT DOES THIS MEAN FOR PATIENTS? Infertility caused by problems in sperm production affects many men. In some cases, sperm development stops at an early stage inside the testis, resulting in a complete absence of mature sperm in the semen (a condition called azoospermia). This study in mice explored whether immature male germ cells, called spermatocytes, can still be used to produce offspring when they are injected into immature oocytes (egg cells). The researchers tested nine types of mutated mice in which sperm development stops at different stages. They found that spermatocytes that had progressed further in their maturation could resume development and produce healthy offspring, whereas less mature spermatocytes could not. These results suggest that, in the future, injection of spermatocytes might help some men with severe infertility who lack mature sperm. However, this approach is still at an early experimental stage, and many ethical and technical issues need to be carefully addressed before considering this application in humans.

## Introduction

At fertilization in mammals, a totipotent zygote is formed by the fusion of the parental gametes, an oocyte, and a spermatozoon. In the zygote, there are two haploid pronuclei, each derived from the oocyte and sperm, cooperatively ensuring subsequent embryonic development to term. At present, it is broadly recognized that this gamete fusion process can be bypassed by direct mechanical delivery of sperm into the ooplasm, in a process called intracytoplasmic sperm injection (ICSI) (Ogura *et al.*, 2005; Uppangala *et al.*, 2015). In 1988, the first normal offspring after ICSI were born in rabbits (Hosoi, 1988), then in bovines (Goto *et al.*, 1990) and humans (Palermo *et al.*, 1992). Soon after these successes, mice were born using round spermatids, which are haploid spermatogenic cells, by electrofusion or injection (Ogura *et al.*, 1994; Kimura and Yanagimachi, 1995). Round spermatid injection (ROSI) also resulted in the production of normal pups in rats (Hirabayashi *et al.*, 2002), rabbits (Hirabayashi *et al.*, 2009), and humans (Tanaka *et al.*, 2015). In humans, ROSI has thus far not been associated with any significant adverse effects on physical or cognitive development during the first two years after birth (Tanaka *et al.*, 2018). Nevertheless, given its extremely low success rate, its broader clinical application will require further technical optimization. Experimental studies in rabbits have demonstrated a high incidence of aneuploidy in ROSI-derived embryos, in contrast to ICSI-derived embryos (Ogonuki *et al.*, 2005). This discrepancy is attributable to the inability of injected round spermatids to provide functional microtubule-organizing centres (MTOCs), which normally originate from sperm centrioles and are indispensable for faithful chromosome segregation in fertilized embryos (Tachibana *et al.*, 2009). In mice, ROSI-derived embryos have also been reported to exhibit epigenetic abnormalities (Kurotaki *et al.*, 2015; Sakamoto *et al.*, 2022; Wang *et al.*, 2022).

In mice, attempts were also made to fertilize oocytes using primary spermatocytes, which are male germ cells before meiosis. To use primary spermatocytes as substitute gametes, their chromosomes should undergo two meiotic divisions within oocytes to form a haploid set of paternal chromosomes. This can be achieved by injecting the primary spermatocyte nucleus into immature germinal vesicle (GV) to metaphase I (MI) oocytes or mature metaphase II (MII) oocytes, with the latter requiring the secondary transfer of the spermatocyte-derived nucleus into another fresh MII oocyte (Kimura *et al.*, 1998; Ogura *et al.*, 1998). In mice, the embryos thus reconstructed with primary spermatocytes had biparental diploid chromosomes and finally developed to normal offspring, but with very low birth rates (1–4%) (Kimura *et al.*, 1998; Ogura *et al.*, 1998; Miki *et al.*, 2006). Chromosomal analysis of the oocytes injected with primary spermatocytes suggested that the major cause of the low developmental efficiency following primary spermatocyte injection was chromosomal aberrations that largely occurred during the first meiotic division (meiosis I) (Kimura *et al.*, 1998; Ogura *et al.*, 1998; Miki *et al.*, 2006). Thus, because of the high incidence of chromosomal aberrations, we do not know the exact potential of the spermatocyte genome to support embryonic development as the paternal genome.

Recently, Kyogoku and Kitajima (2017) reported that oocyte meiotic divisions are inherently error-prone because of the large ooplasm. They showed that a reduction in the size of GV oocytes ensured well-regulated chromosomal behaviour during meiosis I. We applied this technique to spermatocyte injection to see whether the chromosomal behaviour of spermatocyte-injected oocytes could be improved and whether the birth rates could increase. When primary spermatocytes were injected into half-sized MI oocytes and allowed to undergo meiosis (maturation) *in vitro*, the proportion of MII oocytes with normal chromosomal constitution increased from 2% (control) to 21% (Ogonuki *et al.*, 2022). After replacement of the cytoplasm with fresh MII cytoplasm and artificial oocyte activation, a birth rate as high as 19% was achieved (nearly 20-fold improvement vs 1% in control) (Ogonuki *et al.*, 2022). This result has two implications. First, the primary spermatocyte genome is already fully competent as a male gamete, and second, at least some of the male meiotic apparatus can be substituted by the female meiotic apparatus.

Importantly, the spermatocyte injection technique using half-sized oocytes enabled us to produce normal offspring from azoospermic male mice carrying a spermatocyte arrest mutation (Ogonuki *et al.*, 2022). As it is known that a significant proportion of spermatogenic arrest may occur at the primary spermatocyte stage in humans (Enguita-Marruedo *et al.*, 2019), spermatocyte injection might be an option to treat human male-factor infertility in the future. Indeed, azoospermia by the *Stx2* mutation in mice, which we rescued by spermatocyte injection, is one of the infertility factors causing spermatocyte arrest in humans (Nakamura *et al.*, 2018). Mouse and human *Stx2*/*STX2* mutations exhibit a common phenotype of spermatocyte syncytial formation that probably persists until the last stage of prophase I. By contrast, we could not rescue azoospermic males deficient in *D1Pas1* (Ogonuki *et al.*, 2022). D1Pas1 is a mouse autosomal DEAD-box RNA helicase expressed predominantly in the testis, and its deficiency causes spermatocyte arrest at the late pachytene stage by unknown causes (Inoue *et al.*, 2016). Therefore, whether spermatocyte arrest mutations can be rescued may depend on the stage of the spermatocyte meiotic arrest.

In this study, we sought to identify which stages of spermatocyte arrest can be rescued by injection into MI oocytes. For this purpose, we tested several mutant mice that show spermatocyte arrest at different stages. The most advanced spermatocyte stage in each mutant strain of mice was determined by immunocytochemical analysis of chromosome spread preparations of isolated spermatocytes. The results of this study will identify a meiotic stage in which the female meiotic mechanism can (or cannot) substitute for the male mechanism. The results obtained in this study will help to deepen our understanding of the commonalities and differences of meiosis in males and females. In a practical sense, our study will provide important information for determining whether or not spermatocyte injection could be applied to treat human infertility caused by spermatocyte arrest.

## Materials and methods

### Ethical approval and animal housing

The animal experiments described here were approved by the Animal Experimentation Committee of the RIKEN Tsukuba Institute (T2024-EP014) and were performed in accordance with the committee’s guiding principles. All animal handling procedures complied with the ARRIVE guidelines. The mutant mice used in this study are listed in Table 1. Males from mutant strains and the control C57BL/6 strain (Japan SLC, Inc., Shizuoka, Japan) at 4–26 weeks of age were used for the collection of spermatogenic cells. Female B6D2F1 mice (Japan SLC, Inc.) at 9–12 weeks of age were used for the collection of oocytes. Female mice of the ICR strain (CLEA Japan Inc., Tokyo, Japan) at 9–12 weeks of age were used as recipients in embryo transfer experiments. All mice were maintained under a specific-pathogen-free condition, provided with water and commercial laboratory mouse chow *ad libitum*, and housed under controlled lighting conditions (daily light period, 07:00–21:00).

**Table 1.** The mutant mouse strains with spermatocyte arrest used in this study.

Class	Name of mutant gene/protein	Genetic background	Generation of the mutant strain	Function	Source of the mutant strain	Phenotype of females	References
1	*Dynlrb2*/Dynein light chain loadlock-type2	C57BL/6N	CRISPR/Cas9	Dynein light chain roadblock-type-2 regulates a male meiosis dynein light chain that is indispensable for spindle formation in meiosis I.	Generated in this study	Fertile	He *et al.* (2023)
	*Ccna1*/Cycline A1	C57BL/6N	CRISPR/Cas9	A member of the mammalian A type cyclins. Necessary to control entry into metaphase I of spermatogenesis.	Generated in this study	Fertile	Liu *et al.* (1998)
	*Stx2*/Syntaxin 2	C3HeB/FeJ	ENU mutagenesis	A member of the SNARE family. Necessary for intracellular transport and maintenance of intracellular bridges in spermatogenesis.	The Jackson Laboratory and Okayama University	Fertile	Akiyama *et al.* (2008); Fujiwara *et al.* (2013)
	*Exoc1*/Exocyst complex component 1*	C57BL/6J	CRISPR/Cas9	A member of the Exocyst complex. Necessary for normal intracellular vesicle transport and cytokinesis of spermatocytes.	University of Tsukuba	Infertile (Disruption of oocyte follicle growth)	Osawa *et al.* (2021); Nguyen *et al.* (2025)
2	*Kctd19/P*otassium channel tetramerization domain containing 19	Mixed (B6D2F1)	CRISPR/Cas9	A member of potassium channel tetramerization domain (KCTD) family protein. Necessary for meiotic prophase completion during spermatogenesis.	RIKEN (RBRC11037)	Fertile	Oura *et al.* (2021); Horisawa-Takada *et al.* (2021)
	*Emi2 (early meiotic inhibitor 2), Fbxo43*/F-box protein 43	C57BL/6N	CRISPR/Cas9	An inhibitor of the Anaphase-Promoting Complex/Cyclosome (APC/C). Necessary for normal meiosis I progression.	Generated in this study	Infertile (chromosome segregation errors)	Gopinathan *et al.* (2017)
3	*D1pas1/*DNA segment, Chr 1, Pasteur Institute 1	C57BL/6N	ESC homologous recombination	Testis-specific autosomal DEAD-box RNA helicase. Molecular function remains unknown.	RIKEN (RBRC05432)	Fertile	Vong *et al.* (2006); Inoue *et al.* (2016)
	*Setx/*Senataxin	C3HeB/FeJ	Spontaneous mutation	A DNA/RNA helicase involved in transcription, RNA processing, and DNA maintenance.	The Jackson Laboratory	Subfertile (age-dependent)	Becherel *et al.*(2013); Fujiwara *et al.* (2023)
	*Rnf212(Repro57)/*Ring finger protein 212^**^	C3HeB/FeJ	ENU mutagenesis	Necessary for meiotic recombination and crossover.	RIKEN (RBRC09687)	Infertile	Reynold *et al.* (2013); Fujiwara *et al.* (2015); Sono *et al.* (2024)

Class I: mutants that had spermatocytes at the late diplotene stage; Class II: mutants that had spermatocytes at the early, but not late, diplotene stage; Class III: mutants that had pachytene spermatocytes, but not diplotene spermatocytes.

ESCs, embryonic stem cells.

* *Exoc1* mutant spermatocytes were not analysed in this study because they showed the phenotype exactly similar to that of the *Stx2* mutant (Ogonuki *et al.*, 2022).

** *Rnf212* mutant mice had diplotene spermatocytes, but most were aneuploid.

### Collection of oocytes

**Figure 1.**
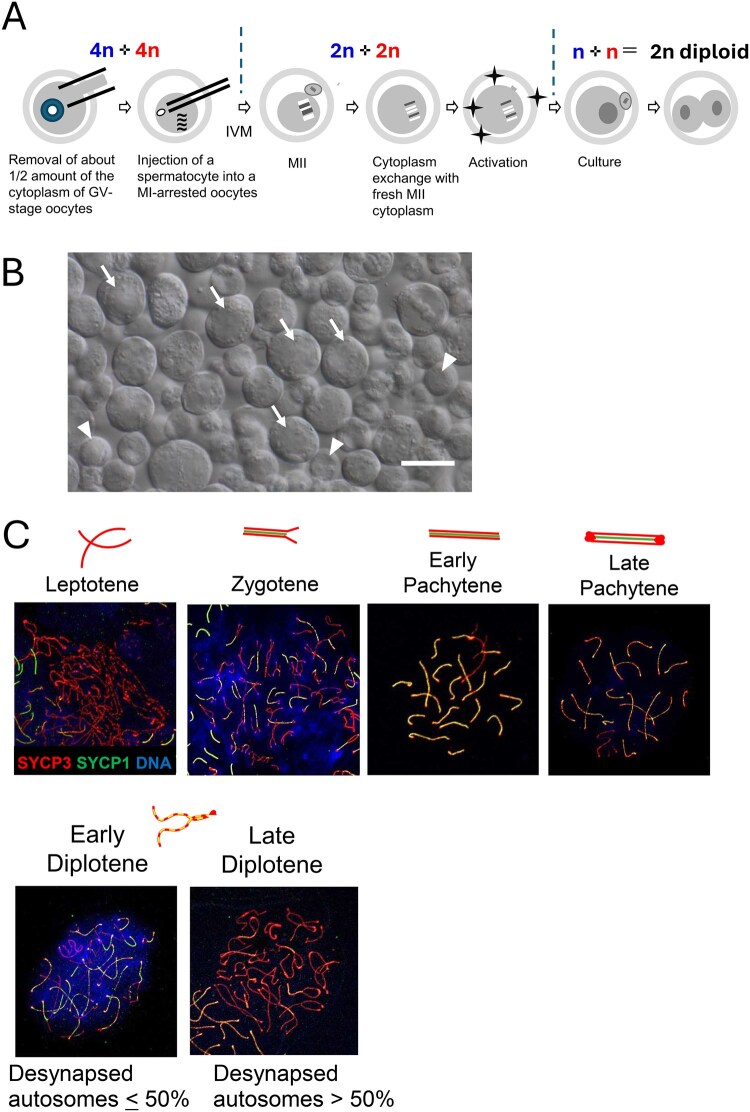
**Production of diploid embryos using primary spermatocytes.** (**A**) Chromosomes from primary spermatocytes injected into immature oocytes undergo two meiotic divisions in synchrony with the meiotic divisions of the host oocyte chromosomes. (**B**) Spermatogenic cell suspension prepared from C57BL/6 testicular seminiferous tubules. In this suspension, elongated spermatids and spermatozoa were removed as aggregates by pronase treatment (Ogura and Yanagimachi, 1993). Primary spermatocytes are the largest cells (arrows), whereas round spermatids are the smallest (arrowheads). Scale bar, 20 µm. (**C**) Identification of the stage of prophase I spermatocytes by immunostaining of chromosome spreads. The chromosome morphology together with the staining patterns of SYCP1 and SYCP3 enabled precise stage classification. Diplotene spermatocytes were further subdivided into ‘early’ and ‘late’ stages based on the proportion of desynapsed autosomes lacking SYCP1 signals. Desynapsed regions were visualized as SYCP3-positive (red) but SYCP1-negative areas. See Supplementary Fig. S1 for higher magnifications. GV, germinal vesicle; MII, metaphase II.

Female B6D2F1 mice were injected with 7.5 IU of equine chorionic gonadotropin (eCG, ASKA Pharmaceutical, Tokyo, Japan). At 44–48 h after injection, fully grown oocytes at the GV stage were collected from large antral follicles and released into M2 medium supplemented with 150 µg/ml dibutyryl-cAMP (dbc AMP, Merck KGaA, Darmstadt, Germany). After being freed from cumulus cells by pipetting, oocytes were cultured for at least 1 h in MEM (Merck KGaA) supplemented with 50 µg/ml gentamicin, 0.22 mM Na-pyruvate, 1 µg/ml EGF, 4 mg/ml bovine serum albumin (BSA) (mMEM), and 150 µg/ml dbc AMP at 37°C in an atmosphere of 5% CO_2_ in humidified air until micromanipulation. We also prepared enucleated MII oocytes for transfer of MII karyoplast containing spermatocyte chromosomes (Fig. 1A). MII oocytes were collected from B6D2F1 females injected with eCG (Nippon Zenyaku Kogyo Co., Ltd, Kouriyama, Japan) and human chorionic gonadotropin (hCG, ASKA Pharmaceutical) and freed from cumulus cells by treatment with 0.1% bovine testicular hyaluronidase. Their MII chromosomes were removed together with spindles in Hepes-buffered CZB containing 7.5 µg/ml cytochalasin B, as in nuclear transfer experiments (Ogura, 2017).

### Collection of primary spermatocytes

Spermatogenic cells were collected from the testes of wild-type (C57BL/6) and mutant males as listed in Table 1. They were isolated by a mechanical method as reported (Ogura and Yanagimachi, 1993; Ogura *et al.*, 1996). Briefly, testes were placed in erythrocyte-lysing buffer (155 mM NH_4_Cl, 10 mM KHCO_3_, 2 mM EDTA; pH 7.2) (Ogura and Yanagimachi, 1993). After removing the tunica albuginea, the testes were transferred into a cold (4–10°C) Dulbecco’s phosphate-buffered saline (PBS) supplemented with 5.6 mM glucose, 5.4 mM sodium lactate, and 3 mg/ml BSA (GL-PBS) (Ogura and Yanagimachi, 1993; Ogura *et al.*, 1996). The seminiferous tubules were cut into small pieces using a pair of fine scissors and pipetted gently to allow spermatogenic cells to be released into the medium. The cell suspension was filtered through a 38-µm nylon mesh and washed twice by centrifugation (200 × *g* for 4 min). After gentle washing, cells were resuspended in GL-PBS and stored at 4°C until microinjection.

### Chromosome spread preparations and immunocytological analysis of spermatocytes

Chromosome spreads were prepared from spermatocytes as previously described (Anderson *et al.*, 1999; de Boer *et al.*, 2009; Milano *et al.*, 2019). Briefly, testes were decapsulated in hypotonic extraction buffer (HEB: 30 mM Tris, pH 8.2, 50 mM sucrose, 17 mM trisodium citrate dihydrate, 5 mM EDTA, 0.5 mM DTT, and 0.5 mM PMSF). Seminiferous tubule fragments were minced in 100 mM sucrose and then fixed onto slides with 1% paraformaldehyde containing 0.15% Triton X-100 in humidified chambers. Slides were washed in 1 × PBS with Photo-Flo 200 (Kodak, NY, USA), dried, and processed for immunostaining, or stored at −80°C until use.

For immunostaining, chromosome spreads were washed in 1 × PBS with 0.4% Photo-Flo 200 (Kodak) and 1 × PBS with 0.1% Triton X-100. The slides were blocked in antibody dilution buffer (ADB: 3% BSA, 0.05% Triton X-100, 10% donkey serum in 1 × PBS). Then, they were incubated overnight at room temperature with primary antibodies: rabbit anti-SYCP1 (NOVUS Biologicals, Centennial, CO, USA) diluted 1:500 and mouse anti-SYCP3 (Abcam, Cambridge, UK) diluted 1:1000 in ADB. Slides were washed as previously and incubated for 1 h at 37°C with secondary antibodies; Alexa Fluor 488 donkey anti-rabbit IgG and donkey anti-mouse conjugated with Alexa Fluor 555 (Life Technologies, Carlsbad, CA, USA), both diluted 1:1000 in ADB. Slides were washed in 0.4% Photo-Flo and mounted with Vectashield Antifade Mounting Medium with DAPI (Vector Laboratories, Newark, CA, USA). All slides were imaged on a Nikon C2si confocal microscope and processed using NIS-Elements AR imaging software (Nikon, Tokyo, Japan).

### Micromanipulations

We reconstructed diploid embryos with primary spermatocytes according to the method previously reported (Ogonuki *et al.*, 2022) (Fig. 1A). In brief, to make half-sized oocytes, oocytes at the GV stage were transferred to M2 medium containing 15 µg/ml cytochalasin D (Merck KGaA) and 60 mM NaCl for 10 min at 37°C. All manipulations were performed under an inverted microscope with a Piezo-assisted micromanipulator (PrimeTech, Ibaraki, Japan). The zona pellucida was opened by piezo drilling, and about half of the ooplasmic volume was aspirated with a glass pipette (inner diameter 20–25 µm) at 37°C. The half-sized oocytes were cultured in mMEM containing 7.5 µg/ml cytochalasin D and 40 mM NaCl at 37°C in an atmosphere of 5% CO_2_ in air. About 1–1.5 h later, primary spermatocytes were injected into oocytes that had been arrested at the MI stage by cytochalasin D. Spermatocytes could be identified by their size and morphology (see Results) (Fig. 1B). Because it is difficult to identify the precise stage of live spermatocytes, we questioned whether a correlation existed between stage and size (see Results). After spermatocyte injection, oocytes were washed in mMEM and cultured for 14–17 h until they reached the MII stage. To refresh the cytoplasm, the karyoplasts containing the maternal and paternal MII chromosomes were removed and then fused with fresh enucleated MII oocytes using Sendai virus (HVJ; Ishihara Sangyo Kaisha, Ltd, Osaka, Japan) in Hepes-buffered CZB medium containing 7.5 µg/ml cytochalasin B. After manipulation, oocytes were cultured in CZB medium containing 7.5 µg/ml cytochalasin B for 1 h at 37°C in an atmosphere of 5% CO_2_ in humidified air until complete fusion occurred. Reconstructed oocytes were washed with fresh CZB and activated by culture in Ca^2+^-free CZB medium containing 8 mM SrCl_2_ for 20 min. After washing, the oocytes were cultured in CZB medium for 24 h until the 2-cell stage under 5% CO_2_ in humidified air at 37°C. Control ICSI experiments were performed according to the method previously reported (Ogonuki *et al.*, 2010) using mature oocytes from superovulated B6D2F1 females and epididymal spermatozoa from C57BL/6N males.

### Embryo transfers

Embryos that reached the 2-cell stage by 24 h were transferred into the oviducts of Day 0.5 pseudopregnant ICR strain female mice. On Day 19.5, recipient females were euthanized and their uteri were examined for live foetuses. Their body weight and placental weight were also recorded. Live foetuses were nursed by lactating foster ICR strain mothers. After weaning, they were checked for fertility by mating with ICR mice of the opposite sex.

### Chromosome preparation of oocytes

Spermatocyte-injected oocytes at the MII stage were incubated in 0.5% actinase E (Kaken Pharmaceutical Co., Tokyo, Japan) for 5 min at room temperature to loosen the zona pellucida and then placed in a hypotonic solution (1:1 mixture of 1.2% sodium citrate and 60% fetal bovine serum, FBS; Merck KGaA) for 10 min at room temperature. Chromosome slides were produced using a gradual-fixation/air-drying method (Mikamo and Kamiguchi, 1983). In brief, oocytes were treated with Fixative I (methanol: acetic acid: distilled water = 5:1:4) for 6–8 min and put onto a glass slide with a small amount of Fixative I. The oocytes were treated with Fixative II (methanol: acetic acid = 3:1) for 2 min and followed by treating with Fixative III (methanol: acetic acid: distilled water = 3:3:1) for 1 min. The slides were air-dried under conditions of 50–60% humidity at 22–24°C. For conventional chromosome analysis, the dried slides were stained with 2% Giemsa (Merck KGaA) for 8 min. C-band staining was performed to distinguish between structural chromosome aberrations and aneuploidy.

### Chromosome analysis of offspring by multicolour fluorescence *in situ* hybridization

Spleens were removed under sterile conditions from mice produced by spermatocyte injection. Splenic lymphocytes were isolated from the spleen and incubated in a tissue culture tube at a cell concentration of 1 × 10^6^/ml in RPMI1640 (Nacalai Tesque, Kyoto, Japan) containing lipopolysaccharide (10 µg/ml, Merck KGaA), concanavalin A (3 µg/ml, Nacalai Tesque), 2-mercaptoethanol (50 µM, Nacalai Tesque), and 6% FBS at 37°C under 5% CO_2_ in humidified air for 48 h. Colcemid (KaryoMAX, Gibco Thermo Fisher Scientific K.K., Tokyo, Japan) at a concentration of 0.02 µg/ml was added to the cell suspension for the last 2 h of culture to arrest the cell cycle at metaphase. The cells were centrifuged at 420 × *g* for 5 min and resuspended in 3 ml of a hypotonic solution (0.075 M KCl). Twenty minutes later, 2 ml of Carnoy’s fixative (methanol: acetic acid = 3:1) was added to the cell suspension. Cells were centrifuged at 420 × *g* for 5 min and resuspended in 5 ml of fresh Carnoy’s fixative. Centrifugations and fixations were repeated three times. Chromosome preparations were made using a Hanabi metaphase spreader (ADSTEC, Chiba, Japan). For multicolour fluorescence *in situ* hybridization (FISH) analysis, the chromosome slides were hybridized with 21XMouse (MetaSystems, Altlussheim, Germany) according to the manufacturer’s protocol. Chromosomal DNA was denatured in 2 × saline sodium buffer (SSC) at 70°C for 30 min and then treated with 0.07 M NaOH at room temperature for 1 min. The denatured slides were washed in 0.1 × SSC and 2 × SSC at 4°C for 1 min each step, and dehydrated with a series of 70%, 95%, and 100% ethanol. Multicolour FISH probes were denatured at 75°C for 5 min and applied to the chromosome slides. After hybridization at 37°C for 48 h in a humidified chamber, the chromosome slides were treated with 0.4 × SSC at 72°C for 2 min, washed in 2 × SSC with 0.05% Tween20 (Merck KGaA) at room temperature for 30 s, and rinsed in distilled water. The slides were counterstained with DAPI/Antifade (MetaSystems) and observed using fluorescent microscopy. Fluorescence images were captured using a high-sensitivity digital camera (7 s, SONY, Tokyo, Japan). The images were imported into ChromaWizard software to assign fluorescence colours to each chromosome. Based on these fluorescence colours, the chromosome numbering was determined. Ten metaphase cells per mouse were analysed for karyotyping.

### Statistical analysis

The body weight and placental weight of offspring derived from spermatocytes of each strain were analysed using Welch’s two-tailed *t*-test (Microsoft Excel, Microsoft 365, Microsoft Corporation, Redmond, WA, USA). A probability of *P *< 0.05 was considered statistically significant.

## Results

### Immunocytological analysis of mutant spermatocytes

First, we sought to identify the most advanced stage of spermatocytes existing in the testis from each mutant strain. Primary spermatocytes can be selected from a testicular cell suspension by their morphology, as they can be identified by their large size (15–25 µm in diameter), relatively small nuclear/cytoplasmic ratio, and rough contour of the nuclear membrane (Fig. 1B). Spermatocytes are softer than somatic cells, making their injection easier. The four major stages of prophase I of primary spermatocytes, leptotene, zygotene, pachytene, and diplotene, could be identified by double staining for SYCP1 and SYCP3 of chromosome spread preparations, as previously described (Alavattam *et al.*, 2018; de la Fuente *et al.*, 2021; Hirano *et al.*, 2022). SYCP1 and SYCP3 are components of the synaptonemal complex, a proteinaceous scaffold that forms between homologous chromosomes, connecting them (synapsis) during meiotic prophase I (Fraune *et al.*, 2012). This structure is gradually assembled: synapsis initiates at zygotene, is complete in pachytene, and progressively dissociates during diplotene. Although SYCP3 is a lateral element protein that is localized to both synapsed and unsynapsed chromosomal regions, SYCP1 is a component of the transverse elements that connect the lateral elements in synapsed chromosomal regions (Fig. 1C). Hence, analysis of the distribution of these proteins and shape of the synaptonemal complex during prophase I allows for the staging of immunostained spermatocytes, as previously described (Alavattam *et al.*, 2018; Hirano *et al.*, 2022). Briefly, in zygotene, SYCP3 is distributed along the entire chromosome length, whereas SYCP1 is restricted to the synapsed regions. The pachytene stage is characterized by the full synapsis of autosomes and, hence, colocalization of SYCP1 and SYCP3 signals. The SYCP3 signal becomes stronger at the autosome ends by late pachytene. The SYCP3 signal becomes even thicker in diplotene. At this stage, the synaptonemal complex gradually disassembles. Lateral elements separate at desynapsed regions, where only the SYCP3 signal remains. Spermatocytes with ≤50% of SYCP1 (−) desynapsed autosomes are staged as ‘early diplotene’, whereas those with >50% desynapsed autosomes are staged as ‘late diplotene’ (Alavattam *et al.*, 2018) (Fig. 1C and Supplementary Fig. S1).

**Figure 2.**
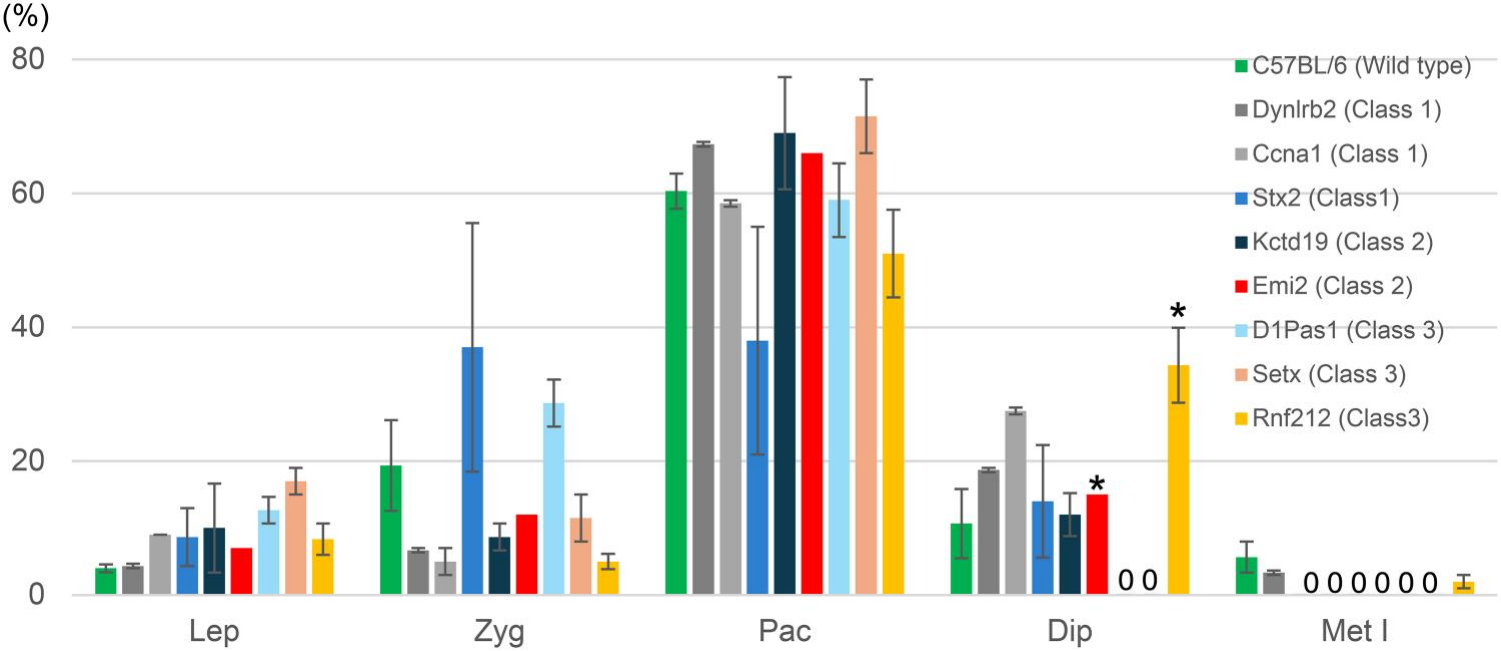
**Distribution of prophase I stages in primary spermatocytes identified by chromosome spread analysis.** Stage distribution of prophase I spermatocytes in testicular cell suspensions from spermatocyte arrest mutants. n = 3, except for *Emi2* (n = 1). Error bars indicate S.D. For the details of the abnormal diplotene chromosomes in *Emi2* and *Rnf212* mutant spermatocytes (asterisks), see Supplementary Fig. S1. Lep, leptotene; Zyg, zygotene; Pac, pachytene; Dip, diplotene; Met, metaphase I.

**Figure 3.**
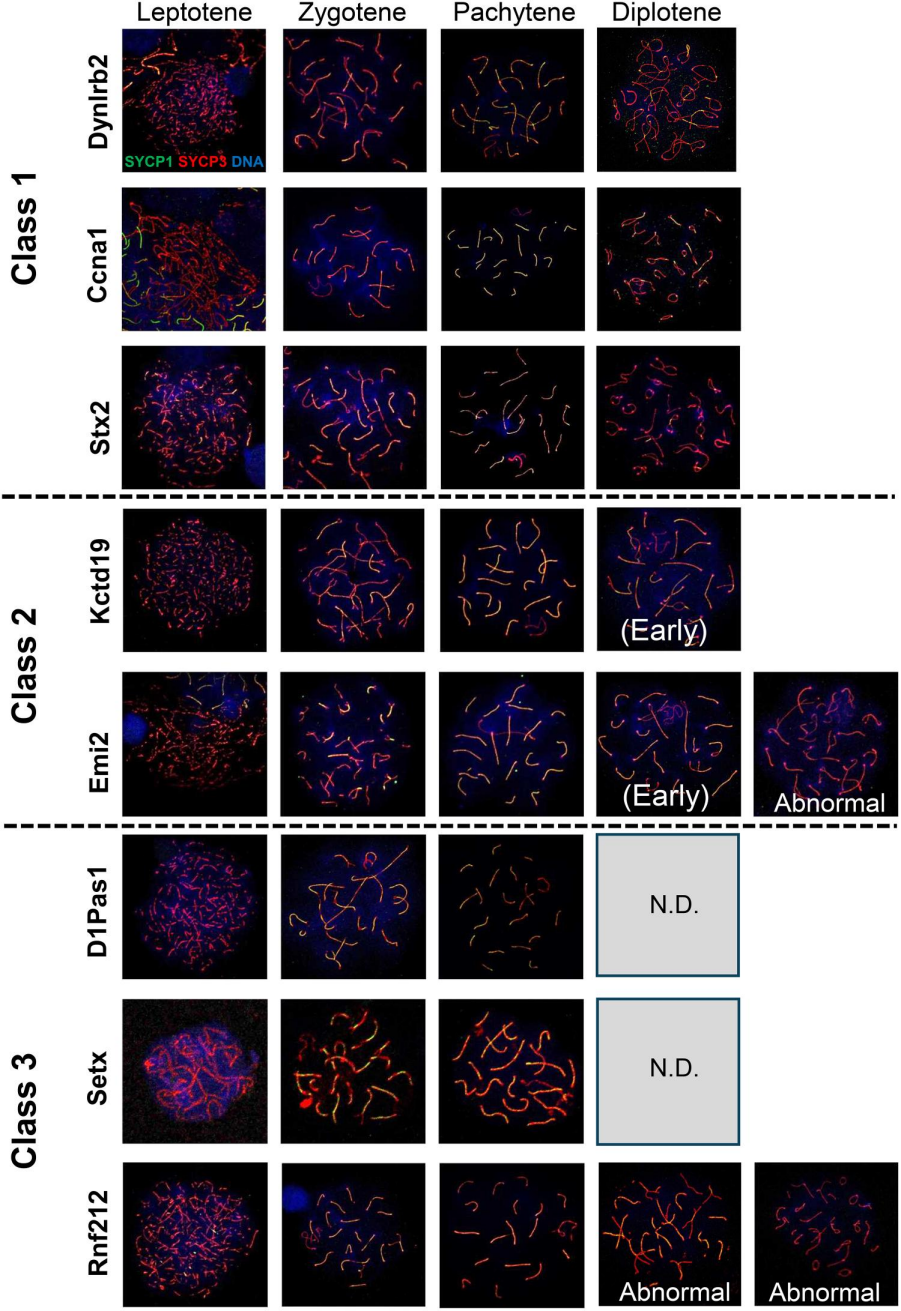
**Representative images of chromosome spreads from mutant mice.** Based on the most advanced stage with normal chromosomal numbers, the mutants can be classified into three classes. Many diplotene spermatocytes from *Rnf212* mutants have abnormal numbers of chromosomes. N.D., not detected.

Analysis of chromosome spread preparations revealed that pachytene spermatocytes represented the largest spermatocyte population in all strains examined, but the most advanced stage varied with the strains (Figs 2 and 3). Accordingly, the spermatocyte arrest mutants could be classified into three categories; mutants that had spermatocytes at late diplotene stage (Class 1; *Dynlrb2*, *Ccna1*, and *Stx2* mutants), those that had early, but not late, diplotene spermatocytes (Class 2; *Kctd19* and *Emi2* mutants), and those that had no diplotene spermatocytes (Class 3; *D1pas1*, *Setx*, and *Rnf212* mutants) (Fig. 3). Spermatocytes from Class 1 mutations rarely showed chromosomal abnormalities whereas those from Class 2 mutations, particularly *Emi2*, had aberrantly separated or damaged chromosomal fragments, leading to increase of the chromosome numbers to more than 20 (Fig. 3, Supplementary Fig. S1). In Class 3 mutations, there were very few normal diplotene spermatocytes. Diplotene spermatocytes from the Class 3 mutations, if any, had abnormal numbers of chromosomes, as typically found in *Rnf212* mutants, due to the crossover failure during the late prophase I stage (Reynolds *et al.*, 2013; Fujiwara *et al.*, 2015) (Figs 2 and 3, Supplementary Fig. S1).

### Diplotene spermatocytes, but not pachytene spermatocytes, supported full-term development following injection into meiotic oocytes

**Figure 4.**
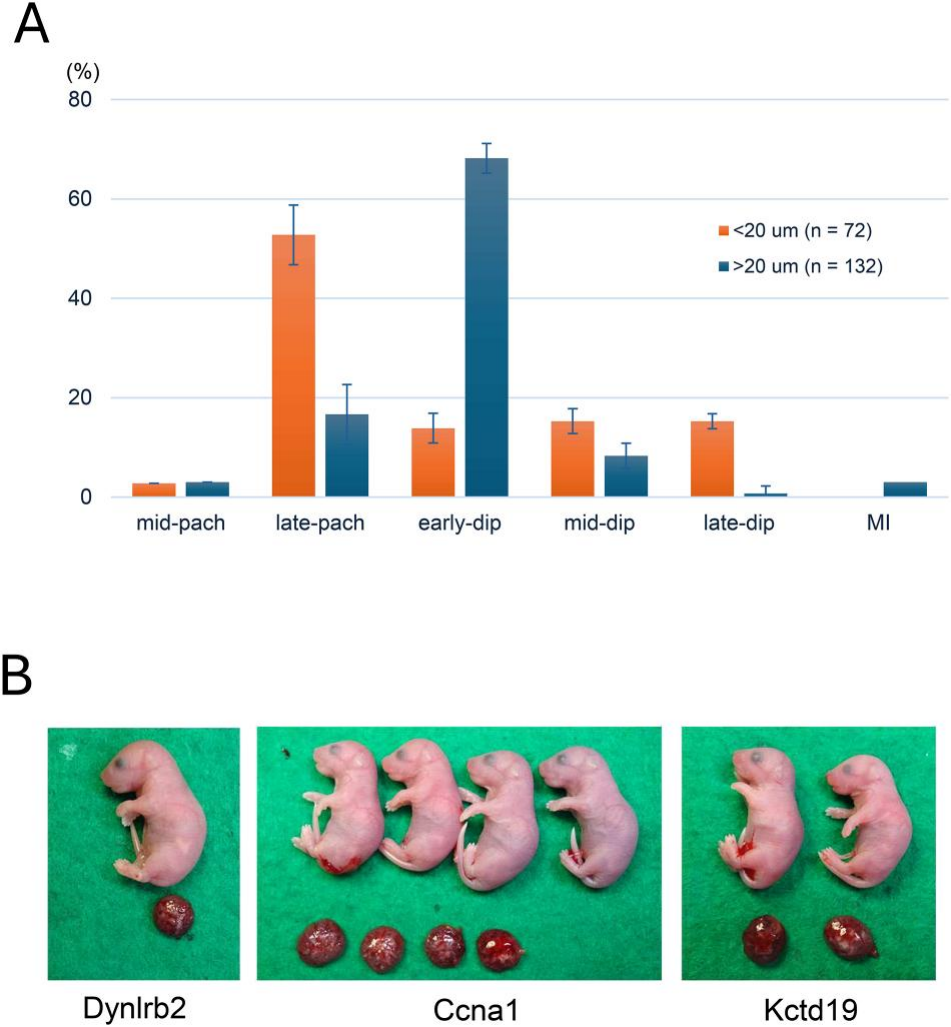
**The relationship between the size and stage of spermatocytes, and mouse pups derived from embryos reconstructed by injection of mutant spermatocytes**. (**A**) Distribution of prophase I spermatocytes classified into two groups based on cell size. n = 2 for each group. Error bars indicate S.D. (**B**) Pups derived from *Dynlrb2*, *Ccna1*, and *Kctd19* mutant spermatocytes appeared morphologically normal and initiated respiration after birth. pac, pachytene; dip, diplotene; MI, metaphase I.

In our spermatocyte injection experiments, we aimed to select primary spermatocytes of the most advanced stage in the testicular cell suspension from each mutant strain. Because we could not identify the exact stage of live spermatocytes picked up for injection, we examined the relationship between the size of spermatocytes and their stages by chromosome spread preparations of normal C57BL/6 spermatocytes. We found that spermatocytes with diameters >20 µm consisted mostly of the early diplotene stage (68%), followed by the late pachytene stage (17%) (Fig. 4A). By contrast, more than half of the smaller size group (17–20 µm) were at the late pachytene stage (53%) (Fig. 4A). Therefore, when we selected the largest spermatocytes in testicular cell suspension, they were most likely diplotene-stage spermatocytes in Class 1 and Class 2 mutants, or late pachytene spermatocytes in Class 3 mutants.

About 27–60% of halved GV-stage oocytes survived spermatocyte injection, depending on the experiment. The rates of injected oocytes that reached the MII stage were relatively consistent, ranging from 54% to 96%. After the exchange of the cytoplasm with that of fresh MII oocytes, the reconstructed oocytes were activated, cultured until the 2-cell stage, and transferred into recipient females (Fig. 1A). The development of reconstructed oocytes *in vitro* and *in vivo* in each mutant strain is summarized in Table 2. Spermatocytes from the *Dynlrb2* and *Ccna1* mutants in Class 1 supported embryonic development to term (Fig. 4B). The remaining two Class 1 mutants, *Stx2* and *Exoc1*, have been rescued by spermatocyte injection, as we previously reported (Ogonuki *et al.*, 2022). Injection with spermatocytes from the *Kctd19* mutant belonging to Class 2 resulted in the birth of normal offspring, indicating that the *Kctd19* deficiency can be rescued by oocyte factor(s) (Fig. 4B). By contrast, use of spermatocytes from the *Emi2* mutant did not result in the production of offspring, although some (12%) of the transferred embryos underwent implantation (Table 2). Two Class 3 mutations, *Rnf212* and *Setx*, could not be rescued by spermatocyte injection even when 46–149 embryos were transferred. Use of another Class 3 mutant, *D1Pas1*, also resulted in no offspring production, as we reported previously (Ogonuki *et al.*, 2022). It is important to note that the embryos reconstructed with Class 3 mutant spermatocytes failed to undergo implantation (0%), suggesting that they suffered from severe chromosomal abnormalities (Table 2).

**Table 2.** Results of injection into oocytes using developmentally arrested spermatocytes from mutant mice.

Class	Name of mutant gene	No. cultured	No. (%) 2-cells	No. transferred	No. (%) implanted	No. (%) offspring	female: male	Fertility of offspring	Chromosome integrity
1	*Dynlrb2*	38	15 (39)	9	3 (33)	1 (11)	1 : 0	All fertile	Not tested
	*Ccna1*	42	29 (69)	29	14 (48)	4 (14)	1 : 3	All fertile	All normal
	*Stx2* *	133	75 (56)	41	19 (46)	5 (12)	4 : 1	All fertile	One female was XO and another had a shortened X chromosome
	*Exoc1* *	96	66 (69)	46	11 (24)	6 (13)	4 : 2	All fertile	One female was XO
2	*Kctd19*	85	66 (85)	66	22 (33)	2 (9)	1 : 1	All fertile	Not tested
	*Emi2*	118	68 (58)	68	8 (12)	0 (0)	–	–	–
3	D1pas1*	65	43 (66)	43	0 (0)	0 (0)	–	–	–
	*Setx*	67	46 (69)	46	0 (0)	0 (0)	–	–	–
	*Rnf212*	191	149 (78)	149	0 (0)	0 (0)	–	–	–

For the category of the Classes, see Table 1.

* Result from Ogonuki *et al.* (2022).

To see the possibility of chromosomal aberrations during meiosis, we analysed the chromosomal constitution in MII oocytes derived from MI oocytes injected with spermatocytes from different mutant strains. Among the oocytes injected with *Ccna1* mutant spermatocytes (Class 1), 10% had the normal MII chromosomes (2n = 40), which was similar to the result of oocytes injected with wild-type spermatocytes (Table 3). By contrast, there were no MII oocytes with the normal chromosomal constitution in the *D1Pas1* mutant (Class 3) group (Table 3). These results are consistent with the developmental ability of embryos derived from these mutant spermatocytes (Table 2). All the pups derived from *Ccna1* mutants (four females and one male) had the normal karyotype, as demonstrated by multicolour FISH analysis (Supplementary Fig. S2).

**Table 3.** Chromosome abnormalities found in metaphase II oocytes after injection with primary spermatocytes.

Strain (male)	Oocytes analysed	Oocytes with normal chr. (%)	Oocytes with abnormal chr. (%)	No. chromosome aberrations (%)		
				predivision	non-disjunction	structural aberrations
C57BL/6 (wild type)*	62	13 (21)	49 (79)	40 (82)	6 (12)	3 (6)
Ccna1 KO	31	3 (10)	28 (90)	25 (89)	3 (11)	3 (11)
D1Pas1 KO	49	0 (0)	49 (100)	48 (98)	1 (2)	10 (20)

* Data from Ogonuki *et al.* (2022). Chr., chromosomes; KO, knock-out.

All the spermatocyte-derived offspring born in this study (11 females and 7 males) appeared phenotypically normal. Their mean birth weights did not differ significantly from those of ICSI-derived control offspring, except for those in the *Stx2* and *Kctd19* mutant groups (Supplementary Fig. S3). Placental weights also showed statistically significant differences only in the *Exoc1* and *Kctd19* mutant groups (Supplementary Fig. S3). All spermatocyte-derived offspring developed into healthy adults and produced progeny after mating with mice of the opposite sex, indicating normal reproductive capacity (Table 2).

## Discussion

**Figure 5.**
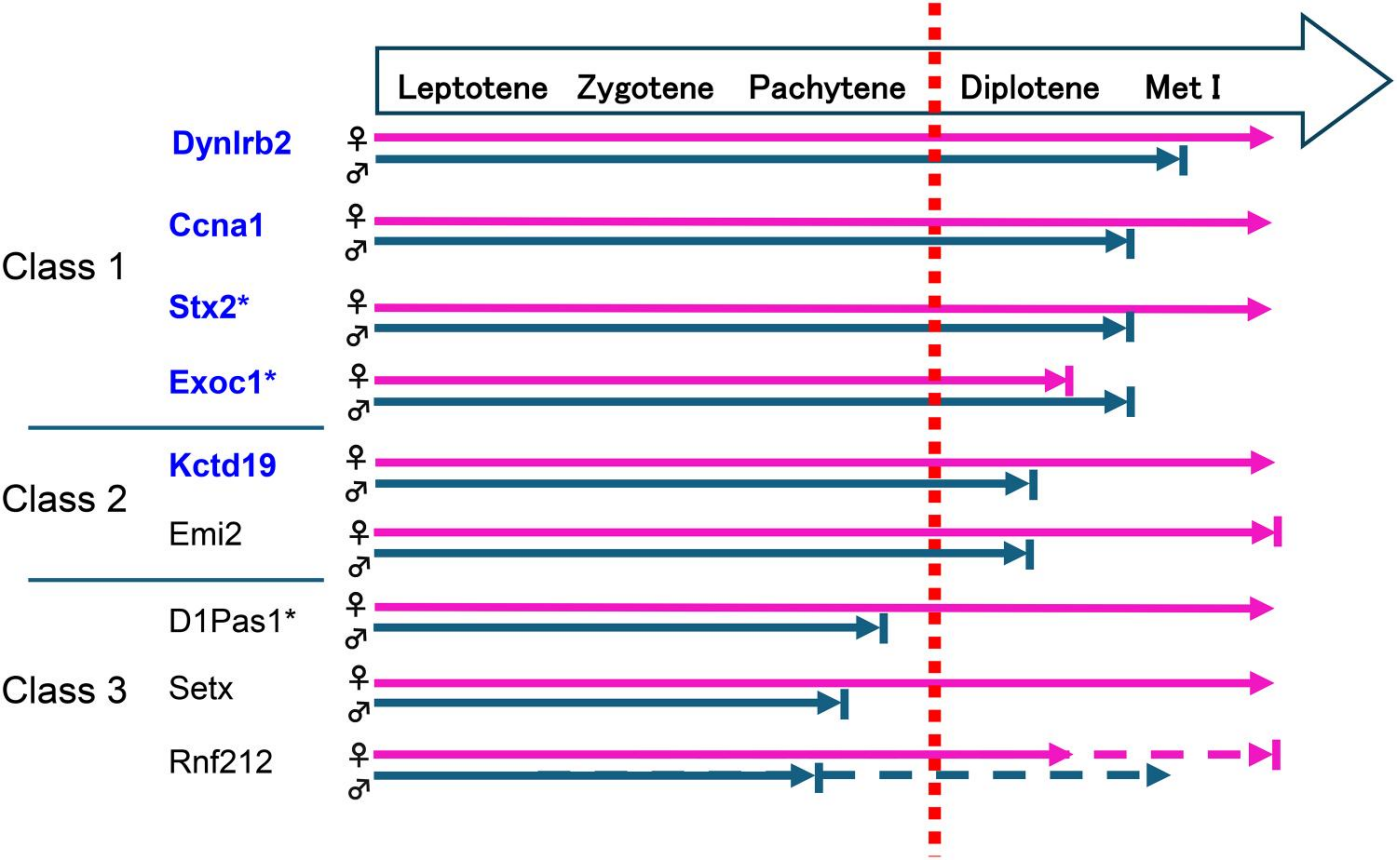
**The stage of spermatocyte arrest and the outcome of spermatocyte injection into immature oocytes**. All Class 1 mutants, which exhibit spermatocyte arrest at late diplotene, were successfully rescued, resulting in the birth of offspring. In Class 2 mutants, characterized by early diplotene arrest, one of the two cases was rescued. By contrast, none of the Class 3 mutants, which arrest at the pachytene stage, could be rescued by spermatocyte injection. Mutant names shown in blue indicate successful rescue by spermatocyte injection. Navy arrows represent spermatocyte development in male mutants, whereas magenta arrows represent oocyte development in corresponding mutant females. Dotted lines indicate developmental pathways associated with chromosomal aberrations. *Reported in Ogonuki *et al.* (2022). Met I, metaphase I.

In this study, we performed a series of spermatocyte injection experiments in mice to rescue azoospermia caused by spermatogenic arrest at different spermatocyte stages. An important implication in this study is that spermatocyte arrest at the late diplotene stage can be rescued by the injection of the spermatocytes into MI oocytes. Despite the promising outcomes in some mutant strains, the number of mutants examined in this study was small. Therefore, further studies across a wider array of spermatogenic arrest models will be necessary to refine our understanding of the factors defining rescue efficiency. Importantly, although one of the two strains with spermatocyte arrest mutation at the early diplotene stage (Class 2) could be rescued, none of the three pachytene arrest mutations (Class 3) could be rescued. These results are summarized in Fig. 5. It is probable that in our experimental system, the meiotic apparatus of the oocyte can substitute the male meiotic apparatus only after the early diplotene stage. It is interesting to note that all five mutants rescued in this study do not cause meiotic abnormalities in mutant female counterparts (Table 1 and Fig. 5). Of them, the *Exoc1* mutation causes infertility in females, but it is not associated with meiotic failure; *Exoc1* mutant females show impaired intra-oocyte trafficking of c-KIT and GDF9 that causes poor follicular development (Nguyen *et al.*, 2025). Thus, although these factors play indispensable roles in the completion of meiosis I in males, they can be omitted under the female meiotic environment.

By contrast, none of the Class 3 mutants, characterized by arrest at the pachytene stage, produced viable embryos following spermatocyte injection. Moreover, no implantation was observed in embryos derived from Class 3 spermatocytes, indicating fundamental chromosomal abnormalities that could not be corrected by the oocyte cytoplasm. These findings suggest a threshold of meiotic progression beyond which spermatocyte nuclei are no longer competent to respond to ooplasmic cues for further development. Consistent with this, no MII oocytes with normal chromosomes were observed when oocytes were injected with spermatocytes arrested at the pachytene stage (*D1Pas1* mutation). One unresolved issue is the inability of pachytene spermatocytes to proceed through meiosis in MI oocytes. The chromosomes of the MI oocyte are already in the end-stage diplotene and ready for the first meiotic division, whereas the pachytene spermatocytes are not. Therefore, it is most probable that the meiotic drive by the oocyte may force pachytene spermatocyte chromosomes to segregate prematurely, resulting in damage to the spermatocyte chromosomes.

ICSI contributes not only to the understanding of the mechanisms of mammalian fertilization but also to the treatment of male infertility in animals and humans. ICSI is highly effective in the treatment of sperm motility defects and obstructive azoospermia, because spermatozoa that have completed spermiogenesis can be injected. However, non-obstructive azoospermia (NOA), which affects about half of azoospermia patients, includes cases without spermatozoa in the testes. In such cases, ROSI and elongated spermatid injection (ELSI) are among the possible treatments. In such cases, ROSI and ELSI represent potential treatment options. Previous studies have reported that both pregnancy and birth rates are markedly lower with ROSI than with ELSI in humans (Gokalp *et al.*, 2021; Tang *et al.*, 2023), consistent with findings from animal models (Ogonuki *et al.*, 2005, 2010). Because the primary spermatocytes used in the present study were developmentally less advanced than round spermatids, numerous ethical, biological, and technical challenges must be overcome before spermatocyte injection can be applied to humans. Biological challenges may involve both genetic and epigenetic abnormalities, as has been reported for ROSI in animals (see Introduction). Moreover, unlike genetically defined laboratory mice, the extensive genetic diversity in humans makes technical optimization particularly difficult. Nonetheless, our findings raise the possibility that spermatocyte injection into oocytes may offer a future avenue for treating male infertility, particularly in patients with NOA caused by meiotic arrest. According to the literature, meiotic (spermatocyte) arrest accounts for about 10–20% of NOA in humans (Schlegel *et al.*, 1997; Tsai *et al.*, 2012). Identifying patients whose spermatocytes are developmentally competent based on our study could open new therapeutic options.

## Supplementary Material

hoaf067_Supplementary_Data

## Data Availability

The data underlying this article are available in the article and in its online supplementary material.
