# Supplementary material for: Spermatocyte injection into meiotic oocytes rescues diplotene, but not pachytene, arrest in azoospermic mutant mice
Source: Hum Reprod Open. 2025 Oct 22;2025(4):hoaf067. doi: 10.1093/hropen/hoaf067 (PMC12628785; doi:10.1093/hropen/hoaf067)
Supplement: hoaf067_Supplementary_Data [file hoaf067_supplementary_data.zip › Ogonuki_et_al. Supplementary (revised).pdf]

Supplementary Figures

Spermatocyte injection into meiotic oocytes rescues diplotene, but not pachytene, arrest in azoospermic mutant mice

Narumi Ogonuki et al.

This file contains Supplementary Figures S1, S2 and S3

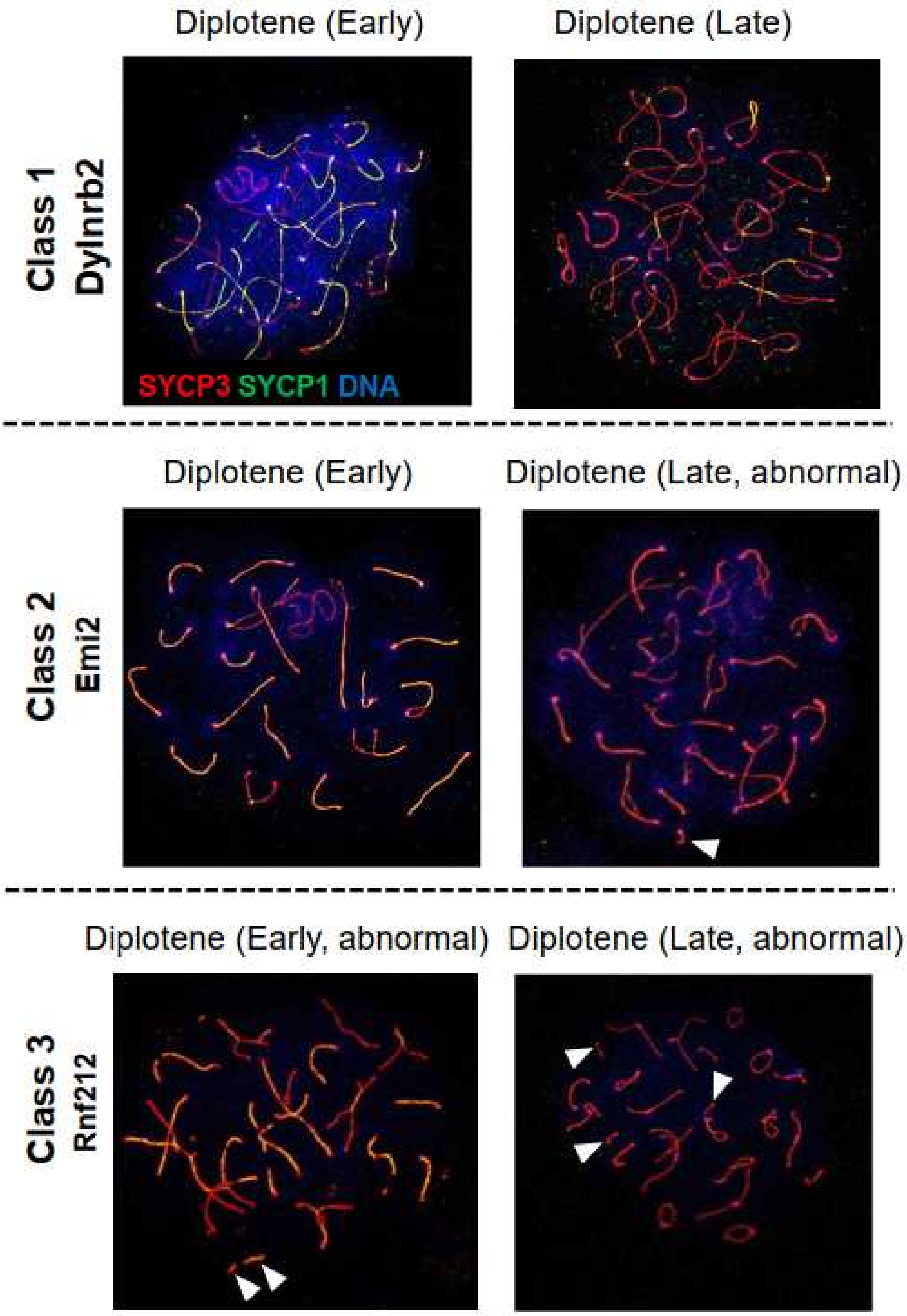
Diplotene (Early)
Diplotene (Late)
Class 1
Dylnrb2
SYCP3 SYCP1 DNA
Diplotene (Early)
Diplotene (Late, abnormal)
Class 2
Emi2
Diplotene (Early, abnormal)
Diplotene (Late, abnormal)
Class 3
Rnf212

Supplementary Fig. S1. Chromosome spread preparations of diplotene spermatocytes (enlargement of Fig. 2). Diplotene spermatocytes can be classified into “early” and “late” stages. Spermatocytes with $\leq$50% of SYCP1 (-) desynapsed autosomes are staged as “early diplotene,” whereas those with >50% desynapsed autosomes are staged as “late diplotene.” Class 1 mutant (*Dynlrb2*) has normal early- and late-stage diplotene spermatocytes, whereas Class 2 mutant (*Emi2*) has only early-stage diplotene spermatocytes. Late-stage diplotene spermatocytes, if any, show abnormal chromosomes. Class 3 mutant spermatocytes arrest development at the pachytene stage, but in *Rnf212* mutant, there is a small number of abnormal diplotene spermatocytes with chromosomal aberrations. White arrowheads indicate chromosomal fragments.

(40,

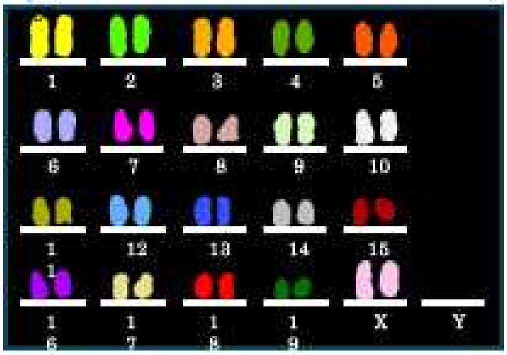

Normal female

(40,

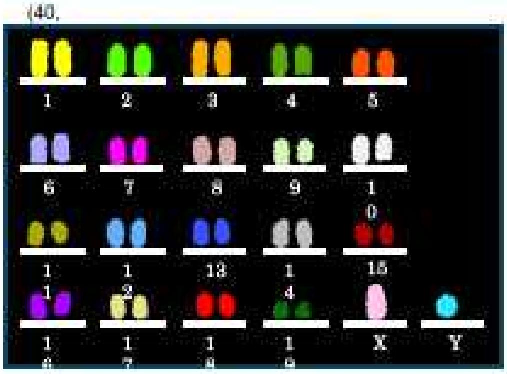

Normal male

(40,

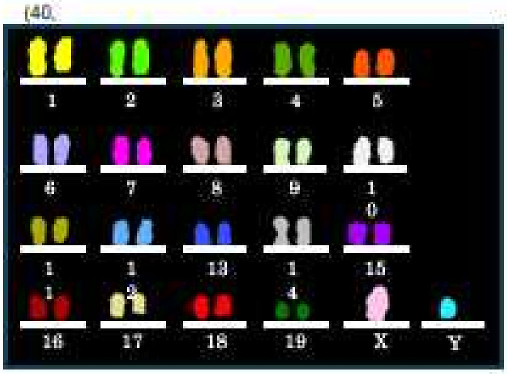

Normal male

(40,

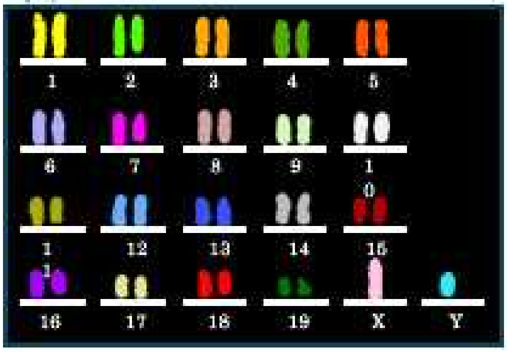

Normal male

Supplementary Fig. S2. Chromosomal multicolor FISH analysis of the offspring derived from *Ccna1* mutant spermatocytes. All offspring (one female and three males) have normal chromosomes.

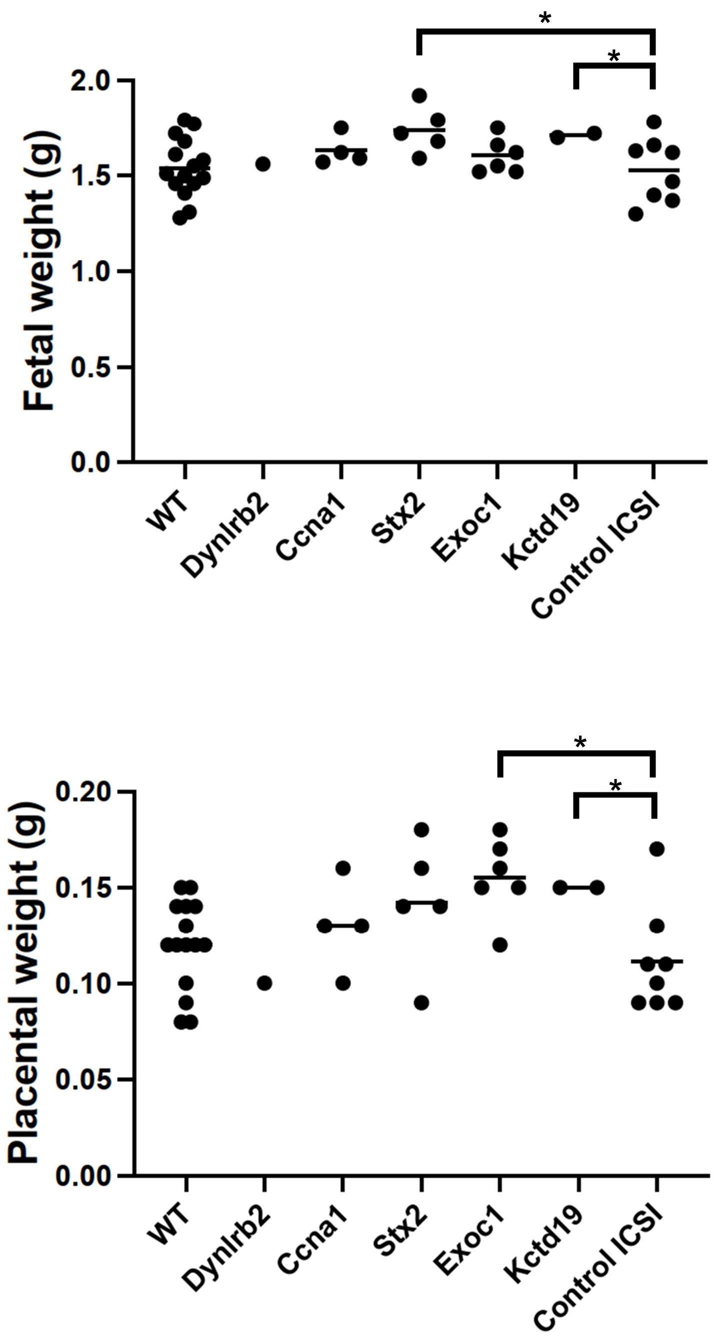

Supplementary Fig. S3. Body weight and placental weight of offspring derived from spermatocytes of different mutations. The body weight and placental weight of offspring derived from spermatocytes of each strain were analyzed using Welch's two-tailed t-test. *$P < 0.05$ compared with control ICSI-derived offspring.
